# Long-term memory reorganization of navigational episodes

**DOI:** 10.1038/s41562-026-02472-x

**Published:** 2026-05-18

**Authors:** Deetje Iggena, Thereza Schmelter, Patrizia M. Maier, Khaled Reguieg, Carsten Finke, Kristian Hildebrand, Christoph J. Ploner

**Affiliations:** 1 Department of Neurology, Charité-Universitätsmedizin Berlin, Berlin, Germany; 2 Berlin School of Mind and Brain, Humboldt-Universität zu Berlin, Berlin, Germany; 3 Berlin Institute of Health at Charité-Universitätsmedizin Berlin, Berlin, Germany; 4 Berlin University of Applied Sciences, Berlin, Germany

**Keywords:** Cognitive neuroscience, Spatial memory, Human behaviour, Consolidation, Long-term memory

## Abstract

During navigation, the brain builds representations of self-motion and of environmental information for future action. The classic view suggests that these representations consolidate and eventually stabilize. However, there are no data on their fate at extended memory delays. Here we investigated memory of real-world navigational episodes across memory delays of up to three decades. We show that memory of the spatial aspects of these episodes do not achieve a stable state but rather continue to transform for many years. Our data suggest that at any given point in time, spatial memory of navigational episodes is a changing combination of episode-independent schematic information and several interacting spatial representations directly related to a navigational episode, which may show distinct temporal trajectories. Consistent with recent accounts of memory reorganization, we further show that neither current theories of systems consolidation nor classic models of forgetting fully explain spatial memory performance at extended memory delays.

## Abstract (web summary)

In this Article, Iggena et al. show that memories of real-world navigational episodes do not simply fade but are continuously reshaped over decades through shifting contributions of episode-related spatial representations and episode-independent spatial schemas.

## Main

When we navigate through an environment, we form representations of spatial information in memory. This information includes the paths we travel, the landmarks we encounter and the spatial relationships between these landmarks and ourselves^1–4^. These spatial memory representations facilitate future behaviour in previously visited locations, for example, to find food or shelter. Everyday experience suggests that navigational episodes are rarely remembered in their entirety over extended periods. Instead, several details fade, whereas other aspects remain salient, thus suggesting that long-term spatial memory of navigational episodes may shift from a detailed cognitive map to a more gist-like spatial representation^5^. However, although the transformative nature of memory consolidation has been stressed repeatedly^6–9^, only few studies have investigated transformation of human spatial memory across real-life delays so far^5,10^.

For natural environments, memory of spatial navigational episodes mostly occurs against the backdrop of semantic knowledge and patterns extracted from previous experiences that shape our expectations and guide actual behaviour^2,5^. For example, when we navigate a city, we expect the town hall to be in the centre of the city; when we go grocery shopping, we expect the commodities of a supermarket to be organized in semantic clusters; when we go hiking, we expect a valley behind a mountain and so on. During individual navigational episodes, these spatial reference templates (schema) are combined with navigation-related information from a first-person perspective (egocentric) and a bird’s-eye perspective (allocentric). Studies suggest that memory of these representational modes relies on at least partially dissociable neural substrates^5,11,12^. Within this framework, episodic memory can be regarded as a superordinate system that hierarchically integrates egocentric and allocentric representations into a coherent spatiotemporal framework.

There is currently sparse evidence whether the relative contributions of egocentric and allocentric representations and their substrates change with extended memory delays and how they interact with previous knowledge. For example, a functional magnetic resonance imaging (fMRI) study on mental navigation in large scale environments showed that involvement of the hippocampus decreases within a year when an environment is highly familiar and is navigated frequently after initial acquisition^13^. A further study found that the involvement of the hippocampus and retrosplenial cortex during a memory-guided navigation task depended on whether the environment was familiar for at least 2 years or newly learned^14^. Conversely, a recent study suggests that the relative contributions of hippocampus and extrahippocampal regions to remote spatial memories are less dependent on the length of the memory delay but rather are dependent on task demands such as the necessity to make precise spatial judgements^15^. Moreover, for other memory domains, previous research found changes in memory representations that were between 2 weeks and 2 years old but no changes for hippocampal and cortical representations of memories that were 2 or 12 years old, thus suggesting that memory consolidation and transformation may be limited to a few years after encoding^16^.

Here, we investigated the spatial aspects of memory of real-world navigational episodes across extended memory delays. Former visitors of a zoo in Berlin were tested for their memory of the spatial layout of the zoo by using immersive virtual reality (VR) and two-dimensional (2D) maps. The delay between participants’ last navigational episode in the zoo and testing varied between 6 days and three decades, thus providing a unique opportunity to investigate memory delays that go far beyond previous studies of spatial memory consolidation. Visitor performance was compared with zoo-naive control subjects. We investigated whether egocentric and allocentric spatial memory representations continue to transform over extended time periods, whether their transformation can be explained with classic power functions of forgetting and how they interact with previous semantic knowledge.

## Results

### Performance in the Berlin Zoo task depends on memory of real-world navigational episodes

**Fig. 1:**
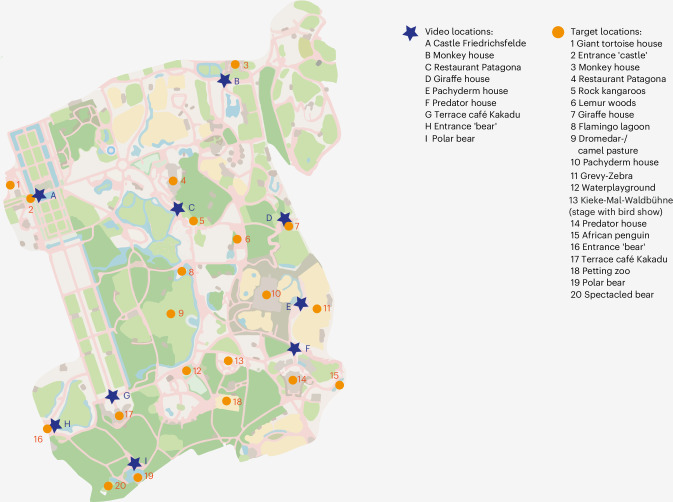
Overview map of the zoo—Tierpark Berlin. The locations of the video recordings are marked with a dark blue asterisk. The locations that served as targets are marked with an orange dot. Map data from OpenStreetMap (https://openstreetmap.org/copyright).

**Fig. 2:**
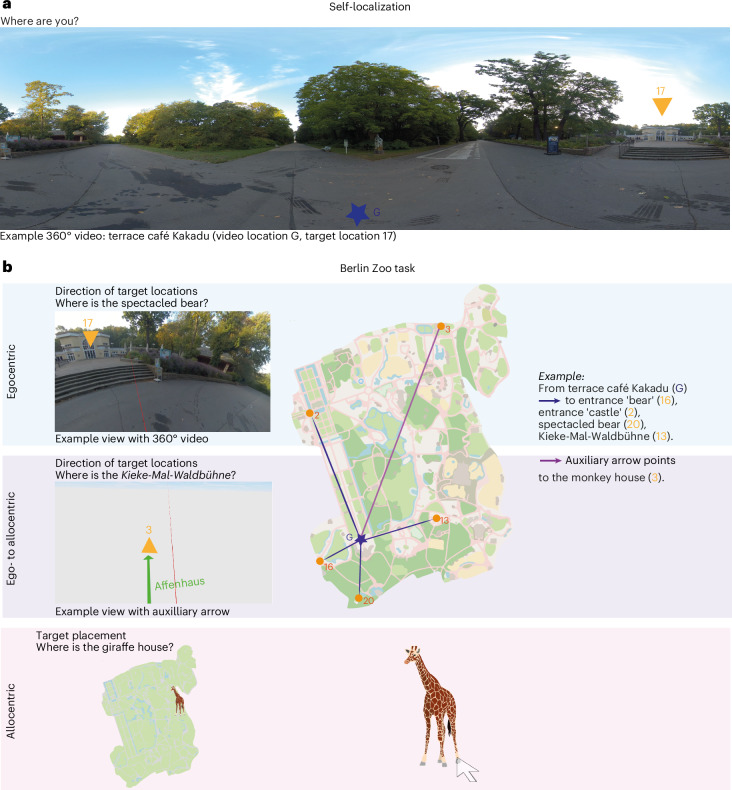
Experimental design. **a**, The self-localization. Participants are asked to locate themselves in the zoo using the environmental information provided in the 360° video. **b**, The Berlin Zoo task. The task consists of three different tasks that depend on egocentric and allocentric spatial representations of the zoo. Two tasks are performed from a first-person perspective in a virtual environment: the direction of target location asks participants to point in the direction of a specific location either by using the environmental information provided in the 360° video (egocentric) or by using an arrow pointing in the direction of a third location (egocentric to allocentric). A third task is performed from a bird’s-eye view on a 2D screen. The target placement asks participants to place a specific location in the correct position on an unlabelled map of the zoo (allocentric). Map data from OpenStreetMap (https://openstreetmap.org/copyright).

We first examined whether performance in the Berlin Zoo task depends on spatial representations acquired during navigation in the zoo and whether previous semantic knowledge contributes to performance (Fig. 1). We therefore tested whether zoo visitors recognized video locations, whether zoo visitors outperform zoo-naive controls on all three tasks of the Berlin Zoo task and whether performance of zoo-naive participants is better than chance (Fig. 2 and Table 1).

**Table 1:** Descriptive participant data. Data are presented as percentage or median and IQR. n.a., not applicable

	Zoo visitors (*n* = 104)	Zoo-naive controls (*n* = 30)
Demographic		
Female/male (%)	78/ 22	73/ 27
Age (years)	34 (26–51)	27 (25–31)
Years of education	17 (16–19)	18.5 (17–19)
Right-/left-/binary handedness (%)	95/ 4/ 1	73/ 20/ 7
Zoo visits		
Number of zoo visits	4 (1.5–10)	n.a.
Time since first visit (days)	6,387 (2,555–11,406)	n.a.
Time since last visit (days)	975 (183–3,665)	n.a.
Age at last visit (years)	31 (21–40)	n.a.

**Fig. 3:**
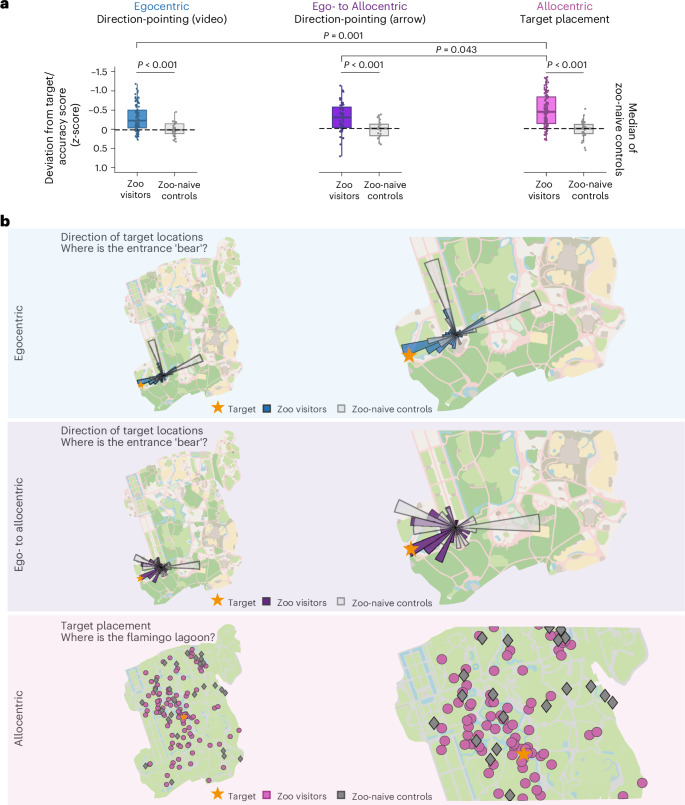
Spatial memory performance of zoo visitors versus zoo-naive controls. **a**, Zoo visitors showed a lower deviation from target locations (*z*-score) and higher accuracy (*z*-score × −1), outperforming zoo-naive controls in all three tasks. Zoo visitors’ performance was better for target placement than for direction-pointing with video or arrow. Box plots are displayed with a centre line as median, Tukey-style whiskers extend 1.5× IQR, dots present individual data points. Zoo visitors: *n* = 104 (direction-pointing (video) and target placement), *n* = 56 (direction-pointing (arrow)); zoo-naive controls: *n* = 30 (direction-pointing (video) and target placement), *n* = 20 (direction-pointing (arrow)). **b**, Example maps for performance in all three tasks. Top: direction-pointing with video. Middle: direction-pointing with arrow. Bottom: target placement on an unlabelled zoo map. Coloured direction indicators and dots indicate zoo visitors; grey direction indicators and rhombuses indicate zoo-naive controls. Map data from OpenStreetMap (https://openstreetmap.org/copyright).

In 77% of cases (interquartile range (IQR) 67–89%), zoo visitors were able to correctly localize themselves using the 360° video (Supplementary Information Section 1.2). The rate of correct responses varied between 17% and 100% across locations and was correlated with zoo visitors’ pointing performance, when video was on display (*ρ*(102) = −0.215, *P* = 0.028, 95% confidence intervals (CI) −0.392 to −0.024). We then compared zoo visitors’ performance with that of zoo-naive controls (Fig. 3a,b and Supplementary Information Sections 1.5 and 1.6). For all three tasks, we found that the average performance of zoo visitors was better than that of zoo-naive controls (direction-pointing (video), *W* = 771, *P* < 0.001, *r* = 0.377, 95% CI −∞ to −0.153; direction-pointing (arrow), *W* = 257, *P* < 0.001, *r* = 0.430, 95% CI −∞ to −0.168; target placement, *W* = 510, *P* < 0.001, *r* = 0.494, 95% CI −∞ to −0.340). These results show that performance in the Berlin Zoo task was neither random nor determined solely by environmental stimuli. Instead, it depended on the availability of a representation of previous navigational episodes in the zoo.

The comparison of zoo visitors performance across all three tasks revealed that memory performance in the target placement task was better compared with direction-pointing with video support and direction-pointing with the aid of an arrow (*χ*^2^(2) = 13.814, *P* = 0.001, *η*^2^ = 0.038; direction-pointing (video) versus direction-pointing (arrow), *z* = 0.590, *P* = 1.0, *r* = 0.041, 95% CI −0.258 to 0.114; direction-pointing (video) versus target placement, *z* = −3.553, *P* = 0.001, *r* = 0.246, 95% CI 0.087 to 0.392; direction-pointing (arrow) versus target placement, *z* = 2.451, *P* = 0.043, *r* = 0.170, 95% CI −0.018 to 0.358). This overall difference in performance across tasks may reflect differences in their sensitivity or in their reliance on schematic knowledge that is independent of specific navigational episodes.

Subsequently, we analysed whether performance in the Berlin Zoo task was additionally driven by environmental information that supports target localization independently of navigational episodes in the zoo. Therefore, we compared the observed error in the performance of zoo-naive controls with the expected error if performance in all three tasks was random. We found that the performance of zoo-naive controls in all tasks was better than chance (direction-pointing (video), *z* = −7.383, *P* < 0.001, *r* = 1.348, 95% CI 1.022 to 1.720; direction-pointing (arrow), *z* = −6.024, *P* < 0.001, *r* = 1.347, 95% CI 0.778 to 1.930; target placement, *z* = −1.82, *P* = 0.037, *r* = 0.332, 95% CI 0.023 to 0.748). Permutation tests indicated no statistically significant difference between the two direction-pointing tasks compared with chance performance (direction-pointing (video) versus direction-pointing (arrow): Δ = −1.54, *P* = 0.344, *d* = −0.256, 95% CI −4.634 to 1.833). However, control participants performed significantly closer to chance in the target placement task than in either of the direction-pointing tasks (direction-pointing (video) versus target placement, Δ = −5.70, *P* < 0.001, *d* = −0.945, 95% CI −8.671 to −2.690; direction-pointing (arrow) versus target placement, Δ = −4.16, *P* = 0.028, *d* = −0.663, 95% CI −7.812 to −0.818). These results suggest that environmental information additionally activated spatial semantic knowledge that may have facilitated inferences about target locations—especially for the two direction-pointing tasks.

Despite the observed differences in overall performance levels, correlation analyses revealed shared variance between tasks. Positive correlations were observed across all task pairs, with strong associations between egocentric and egocentric-to-allocentric (*ρ*(54) = 0.640, *P* < 0.001, 95% CI 0.428 to 0.770) and between egocentric-to-allocentric and allocentric performance (*ρ*(54) = 0.801, *P* < 0.001, 95% CI 0.632 to 0.889) and a moderate association between egocentric and allocentric performance (*ρ*(102) = 0.465, *P* < 0.001, 95% CI 0.275 to 0.624). The correlation between egocentric-to-allocentric and allocentric tasks was significantly stronger than that between egocentric and egocentric-to-allocentric tasks (*z* = 2.627, *P* = 0.009, 95% CI 0.045 to 0.293), which in turn exceeded the correlation between egocentric and allocentric tasks (*z* = 3.343, *P* < 0.001, 95% Cl 0.077 to 0.291). Together, this graded pattern is consistent with partially overlapping spatial reference frames.

### Memory of real-world navigational episodes transforms nonlinearly across decades

**Fig. 4:**
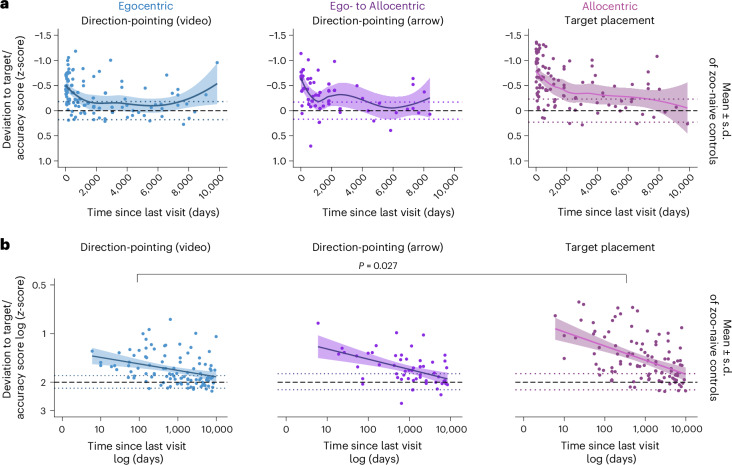
Memory of navigational episodes in the zoo over a period of 6 days to three decades since last visit. **a**, For zoo visitors, data on all three tasks were locally weighted and smoothed for visual inspection. The performance on all tasks was significantly correlated with the time elapsed since the last visit to the zoo. The solid line depicts the regression line; the shaded area indicates the 95% CI. Points represent individual observations. The dashed line indicates mean performance of zoo-naive controls. The dotted lines indicate the first standard deviation above and below the mean performance of zoo-naive controls. **b**, The power regression for all three tasks revealed a decline in memory performance as a function of the time since the last visit to the zoo. The regression slope differed significantly between direction-pointing (video) and target placement. The solid line depicts the regression line; the shaded area indicates the 95% CI. Points represent individual observations. The dashed line indicates mean performance of zoo-naive controls. The dotted lines indicate the first standard deviation above and below the mean performance of zoo-naive controls. Note: *z*-scores were shifted into the positive range before applying a log transformation.

The visual inspection of the *z*-scores indicated a nonlinear relationship between time and spatial memory performance for all three tasks (Fig. 4a). To describe the relationship further, we correlated spatial memory performance with the time elapsed since the last visit to the zoo. For all three tasks, we found that spatial memory performance continuously decreased with increasing time since last visit (Fig. 4a,b and Supplementary Information Section 1.7) (direction-pointing (video), *ρ*(102) = 0.25, *P* = 0.010, 95% CI 0.225 to 0.552; direction-pointing (arrow), *ρ*(54) = 0.370, *P* = 0.004, 95% CI 0.234 to 0.651; target placement, *ρ*(102) = 0.350, *P* < 0.001, 95% CI 0.294 to 0.601). When comparing performance between tasks, we found no differences in correlations (direction-pointing (video) versus direction-pointing (arrow), *z* = −1.521, *P* = 0.128, 95% CI 0.453 to 0.773; direction-pointing (video) versus target placement, *z* = −1.038, *P* = 0.299, 95% CI 0.230 to 0.648; direction-pointing (arrow) versus target placement, *z* = −0.346, *P* = 0.729, 95% CI 0.682 to 0.879).

To investigate the causal relationship between time and spatial memory performance further, we performed a regression analysis of spatial memory performance for all three tasks over time. After using eight different regression models, the Akaike information criterion revealed that a power regression best explained the impact of time since last visit on spatial memory performance in all three tasks (Supplementary Information Section 1.7). After fitting the data with the power model for each task, an interaction model showed that performance was influenced by the time since the last zoo visit, with slopes differing significantly across tasks. This indicates a task-dependent effect of time. An analysis of variance comparing an additive model to the interaction model confirmed a significantly better fit when including the interaction between time since last zoo visit and task (*F*_(2, 256)_ = 3.355, *P* = 0.036, *ꞷ*^2^ = 0.01). Although all tasks showed performance deterioration over time, the rate of decay was greatest in the target placement task and smallest in the direction-pointing with video task (direction-pointing (video), *β* = 0.040, *t*_(256)_ = 3.092, *P* = 0.002, *r* = 0.190, 95% CI 0.015 to 0.066; direction-pointing (arrow), *β* = 0.062, *t*_(256)_ = 3.451, *P* < 0.001, *r* = 0.211, 95% CI 0.027 to 0.099; target placement, *β* = 0.088, *t*_(256)_ = 6.755, *P* < 0.001, *r* = 0.389, 95% CI 0.062 to 0.113). Pairwise comparisons of the estimated regression slopes revealed that memory decay differed between direction-pointing with video and target placement, that is, between the purely egocentric and purely allocentric tasks (direction-pointing (video) versus direction-pointing (arrow), Δ*β* = 0.023, *t*_(256)_ = 1.036, *P* = 0.555, *r* = 0.065, 95% CI −0.030 to 0.076; direction-pointing (video) versus target placement, Δ*β* = −0.048, *t*_(256)_ = −2.590, *P* = 0.027, *r* = 0.160, 95% CI −0.090 to −0.004; direction-pointing (arrow) versus target placement, Δ*β* = −0.024, *t*_(256)_ = −1.077, *P* = 0.529, *r* = 0.067, 95% CI −0.077 to 0.029).

To further characterize task-specific performance trajectories, we conducted pairwise contrasts between the three spatial tasks across an extended time window (6–10,000 days since last zoo visit). For the two pointing tasks (direction-pointing (video) versus direction-pointing (arrow)), we found no significant differences at any time point (*P* > 0.49). Significant differences emerged between direction-pointing with arrow and target placement beginning on day 14 and persisted until day 2447 (6.7 years, *P* < 0.05), suggesting a gradual and sustained divergence between these tasks over time. A robust and consistent difference between direction-pointing with video and target placement was evident across all assessed time points up to day 2,776 (7.6 years, *P* < 0.05), reflecting a stable performance gap between these two conditions.

We subsequently estimated the time point at which the spatial memory performance of zoo visitors converged with that of zoo-naive controls—that is, when task performance would no longer be driven by spatial memory representations of previous navigational experiences but would instead reflect semantic knowledge and logical inference. To estimate this transition point, we selected the most appropriate and interpretable model, a power regression fitted across all tasks. For direction-pointing with video, the average performance of the zoo visitors would be expected to reach the mean performance level of zoo-naive controls after approximately 184 years (Supplementary Information Section 1.8). For the direction-pointing with arrow task, zoo visitors’ average performance would converge with that of zoo-naive controls after about 48.5 years. In the target placement task, a comparable level of performance would be expected to emerge after roughly 98 years.

**Fig. 5:**
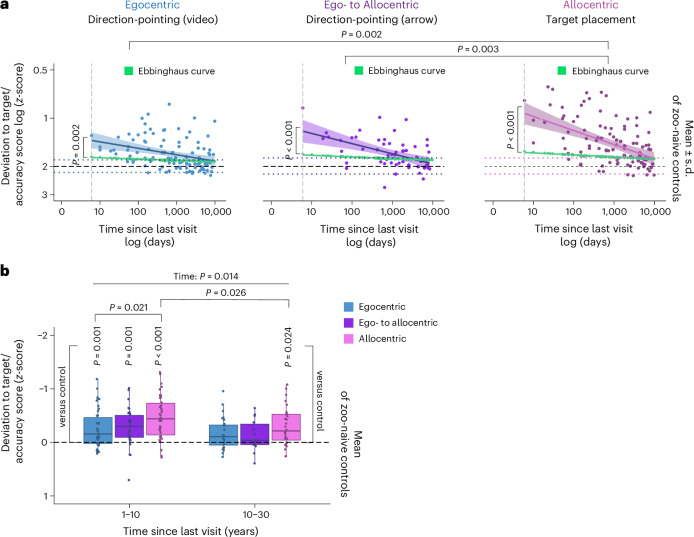
Memory of navigational episodes in the zoo compared with the Ebbinghaus curve and zoo-naive controls. **a**, The temporal trajectories for all tasks were compared with data simulated using the Ebbinghaus equation (6 days to 30 years). Curves were aligned to a common score at the first minute (outside the observation window), on the basis of their respective power regressions. Each task showed a different slope compared with the simulated data, with the most pronounced deviation for target placement, whose slope differed significantly from both pointing tasks. Solid line, regression; shaded area, 95% CI; points, individual observations; vertical dashed line, 8,460th minute; dashed line, zoo-naive control mean; dotted lines, ±1 s.d. of controls. *z* scores were shifted into the positive range before log transformation and calculated per individual query. **b**, The performance of zoo visitors versus controls across two post zoo visit intervals (1–10 years versus 10–30 years). Zoo visitors showed substantial overall retention, with all tasks differing significantly from controls in the first decade. In the later interval, only target placement remained significantly above control levels. A significant main effect of task was found within the early interval, with post hoc tests indicating better performance on direction-pointing (video) versus target placement; this effect was not present in the later interval. Across time intervals, a significant time effect was revealed with post hoc tests indicating a decrease in performance over time for target placement. The dotted line indicates the mean performance of zoo-naive controls. Data presented as box plots, centre line, median; Tukey-style whiskers, 1.5× IQR; dots, individual data points. The 1–10-year interval, *n* = 44 (pointing (arrow), *n* = 31); the 10–30-year interval, *n* = 26 (pointing (arrow): *n* = 13).

As the performance of zoo visitors did not stabilize but rather continued to decrease to the level of zoo-naive controls, the relationship between time and spatial memory performance in our study can equally be conceived in frameworks of consolidation and of forgetting. We therefore investigated whether representations of past navigational episodes show temporal trajectories of forgetting consistent with the highly influential Ebbinghaus curve^17–20^. The investigated model included the predictors time since last visit (centred on minute 1), task (direction-pointing (video), direction-pointing (arrow) and target placement) and data source (real versus simulated data according to Ebbinghaus function) (Fig. 5a). After aligning the curves with the same starting value on a hypothetical first minute for each task, we compared the simulated and real data curves from day 6 to day 10,000 across tasks (three-way interaction time × task × source, *F*_(2, 559)_ = 2.976, *P* = 0.052, *η*^2^ = 0.011). Significant two-way interactions were observed for time × task (*F*_(2, 559)_ = 4.949, *P* = 0.007, *η*^2^ = 0.017), time × source (*F*_(1, 559)_ = 50.233, *P* < 0.001, *η*^2^ = 0.082) and task × source (*F*_(2, 559)_ = 7.743, *P* < 0.001, *η*^2^ = 0.027). Follow-up contrasts revealed that real memory performance declined more steeply than predicted by the Ebbinghaus function across all three tasks (direction-pointing (video), *t*_(559)_ = –3.108, *P* = 0.002, *r* = 0.165, 95% CI −0.134 to −0.045; direction-pointing (arrow), *t*_(559)_ = –3.960, *P* < 0.001, *r* = 0.130, 95% CI −0.136 to −0.031; target placement, *t*_(559)_ = –8.791, *P* < 0.001, *r* = 0.348, 95% CI −0.242 to −0.154). Post hoc comparisons between tasks showed that the difference between real and simulated data was significantly greater for target placement than for either of the direction-pointing tasks, whereas the two direction-pointing tasks did not significantly differ from one another (direction-pointing (video) versus direction-pointing (arrow), *t*_(559)_ = 0.161, *P* = 1.0, *r* = 0.0, 95% CI −0.077 to 0.088; direction-pointing (video) versus target placement, *t*_(559)_ = 3.264, *P* = 0.002, *r* = 0.143, 95% CI 0.034 to 0.184; direction-pointing (arrow) versus target placement, *t*_(559)_ = 3.416, *P* = 0.003, *r* = 0.137, 95% CI 0.032 to 0.197). Together, these results suggest that real-world spatial memory decay deviates from classical forgetting curves and that the extent of this deviation may be task-specific.

To assess whether memory performance continues to transform at extended retention intervals beyond a few years, we conducted direct group comparisons between participants tested 1–10 years after their zoo visit and controls, between participants tested 10–30 years after their visit and controls as well as between the two retention interval groups (1–10 years versus 10–30 years) themselves (Fig. 5b). In both the 1–10-year and 10–30-year intervals, overall zoo visitors’ performance was significantly better than in controls (1–10 years, *W* = 262, *P* < 0.001, *r* = 0.516, 95% CI −∞ to −0.206; 10–30 years, *W* = 220, *P* = 0.002, *r* = 0.376, 95% CI −∞ to −0.064). In the 1–10-year interval, all three tasks differed significantly from control performance (direction-pointing (video), *W* = 373, *P* = 0.001, *r* = 0.377, 95% CI −∞ to −0.098; direction-pointing (arrow), *W* = 129, *P* = 0.001, *r* = 0.489, 95% CI −∞ to −0.161; target placement, *W* = 233, *P* < 0.001, *r* = 0.552, 95% CI −∞ to −0.314). By contrast, in the 10–30-year interval, only the target placement task showed a significant difference (W = 246, *P* = 0.024, *r* = 0.319, 95% CI −∞ to −0.053), whereas both direction-pointing tasks did not (direction-pointing (video), *W* = 311, *P* = 0.287, *r* = 0.223, 95% CI −∞ to 0.20; direction-pointing (arrow), *W* = 110, *P* = 0.934, *r* = 0.092, 95% CI −∞ to 0.087) suggesting a different temporal trajectory for spatial representations even after the first decade. Within the 1–10-year interval, a significant main effect of task was found (*χ*^2^(2) = 7.956, *P* = 0.019, *η*^2^ = 0.045), with post hoc comparisons revealing a significant difference between direction pointing with video and target placement (direction-pointing (video) versus direction-pointing (arrow), *W* = 762, *P* = 1.0, *r* = 0.183, 95% CI −0.090 to 0.225; direction-pointing (video) versus target placement, *W* = 1288, *P* = 0.021, *r* = 0.285, 95% CI 0.059 to 0.40; direction-pointing (arrow) versus target placement, *W* = 856, *P* = 0.185, *r* = 0.100, 95% CI −0.005 to 0.359). No such task effect was found in the 10–30-year group (*χ*^2^(2) = 2.995, *P* = 0.224, *η*^2^ = 0.013), suggesting that task performance differences diminish over time. Across time intervals, we found a significant overall decline in performance (*W* = 391, *P* = 0.014, *r* = 0.263, 95% CI −∞ to −0.038), suggesting ongoing memory transformation beyond the first year after acquisition. Post hoc analyses showed that this effect was driven by a performance decline in the target placement task (*W* = 377, *P* = 0.026, 95% CI −∞ to −0.077), whereas the pointing tasks showed no significant differences between intervals (direction-pointing (video), *W* = 441, *P* = 0.170, 95% CI −∞ to 0.009; direction-pointing (arrow), *W* = 119, *P* = 0.050, 95% CI −∞ to −0.048).

### Memory of real-world navigational episodes also depends on time-independent factors

Further analysis revealed that the time since last visit only explained 11–24% of the observed variance in spatial memory performance (direction-pointing (video) adjusted *R*^2^ value (*R*^2^ (adj.)) 0.101, deviance explained 11%; direction-pointing (arrow) *R*^2^ (adj.) 0.227, deviance explained 24.1%; target placement, *R*^2^ (adj.) 0.186, deviance explained 24.4%; Table 2).

**Table 2:** Table of results

	Direction-ponting (video)	Direction-pointing (arrow)	Target placement
GAM with multiple factors	Orientation to north, *t* = 4.457, *P* < 0.001, linear, ↑ Time since last visit (smoothed and log-transformed), *F* = 2.713, *P* = 0.007, edf = 8.006, nonlinear, ↑ Age at last visit (smoothed), *F* = 4.401, *P* < 0.001, edf = 7.70, nonlinear, ↓ Deviance explained: 58.8% *R*^2^ (adj.) = 0.507	Orientation to North, *t* = 3.867, *P* < 0.001, linear, ↑ Time since last visit (smoothed and log-transformed), *F* = 6.400, *P* < 0.001, edf = 3.440, nonlinear, ↑ Time since first visit (smoothed), *F* = 4.088, *P* = 0.012, edf = 2.419, nonlinear, ↓ Deviance explained: 61.8% *R*^2^ (adj.) = 0.563	Time since last visit (smoothed and log-transformed), *F* = 8.161, *P*< 0.001, edf = 3.142, nonlinear, ↑ Time since first visit (smoothed), *F* = 7.596, *P* < 0.001, edf = 1.829, nonlinear, ↓ Age at last visit (smoothed), *F* = 2.90, *P* = 0.006, edf = 7.792, nonlinear, ↓ Deviance explained: 64% *R*^2^ (adj.) = 0.496
GAM with a single factor	Time since last visit (log-transformed), *t* = 3.531, *P* = 0.002, linear, ↑ Deviance explained: 11% *R*^2^ (adj.) = 0.101	Time since last visit (log-transformed), *t* = 4.140, *P* < 0.001, linear, ↑ Deviance explained: 24.1% *R*^2^ (adj.) = 0.227	Time since last visit (log-transformed), *t* = 5.717, *P* < 0.001, linear, ↑ Deviance explained: 24.4% *R*^2^ (adj.) = 0.237

Best-fitting GAM per task. Only relevant and significant contributing predictors are reported. ↑ indicates positive direction of the relationship, ↓ indicates negative direction of the relationship, edf = estimated degrees of freedom.

We therefore analysed whether additional factors in conjunction with time since the last visit could better explain spatial memory performance of zoo visitors in the Berlin Zoo task. For each task, we tested eight predictors: time since last visit, time since first visit, current age, age at last zoo visit, number of zoo visits, years of education, Santa Barbara Sense of Direction Scale (SBSOD) and orientation towards north during the task. For the direction-pointing with video task, the best generalized additive model (GAM) explained 58.8% of the variance, incorporating time since the last visit (more recent visits were associated with better performance, that is, lower *z*-scores), northward orientation during the task (better orientation was associated with better performance) and age at the time of the last visit (older age at the last visit was associated with better performance). For the direction-pointing with arrow task, the optimal GAM explained 61.8% of the variance, with contributing factors including time since the last visit (more recent visits were associated with better performance), northward orientation (better orientation was associated with better performance) and time since the first visit to the zoo (more distant first visits were linked to better performance). Similarly, for the target placement task, the best GAM accounted for 64% of the variance, with time since the last visit (more recent visits were associated with better performance), time since the first visit (more distant first visits were linked to better performance) and age at the last visit (older age at the last visit was associated with better performance) as the most relevant predictors. The final model presented in Table 2 is the simplest model that best explains spatial memory performance, incorporating only the most significant contributing factors.

## Discussion

We investigated memory of navigational episodes from a real-world setting, a zoo in Berlin, across memory delays of up to three decades. Our results show that zoo visitors’ spatial memory of their navigational experience does not achieve a stable state but rather continues to transform for many years. Our data are consistent with the hypothesis that at any given point in time, memory of navigational episodes is a continuously changing combination of episode-related egocentric and allocentric spatial representations with episode-independent schematic knowledge. These representations show decay rates that do not necessarily follow classic models of forgetting and may follow distinct temporal trajectories, suggesting continuously interacting neural mechanisms that support their maintenance even years after a navigational experience.

Historically, the term consolidation suggests the transition of memory representations from an initial labile to a more stable state. Although initially applied to memory experiments on a timescale of minutes, the term was later also used to explain temporally graded amnesia in patients with temporal lobe damage^6,21^. The seeming preservation of remote autobiographical memories in these patients was compatible with the hypothesis that consolidation at the level of brain systems is a process that takes years and ultimately renders memories resistant even against brain damage. It further suggested that consolidation leads to a progressive shift from hippocampal to extrahippocampal brain regions for storage of memories, a hypothesis that is still under debate^6,8,9,22^. For obvious methodological reasons, it has proven difficult to investigate the time course of systems consolidation experimentally. Therefore, only few studies have addressed the hypothetical years-long process in human participants without brain damage. Most of these studies used episodic detail of autobiographical memories as a variable to assess memory retention at extended memory delays^23–27^. Similar to our results, these studies showed a progressive nonlinear and context-dependent decline of memory up to decades that is not easy to reconcile with the idea of a memory ‘permastore’ that has been postulated for semantic memory^28^. By contrast, in a series of fMRI experiments on normal human subjects recalling autobiographical episodes from up to 10 or 12 years ago, no differences were found between memories that were 2 or 12 years old, thus suggesting that autobiographical memory consolidation may be largely complete by, at most, 2 years after memory formation^16,29^. Only few fMRI studies have investigated consolidation of spatial memory by comparing navigational performance or distance judgements in environments learned years ago. However, it has remained equivocal whether consolidation-related changes in brain activation are mainly driven by time-related factors or the demands of the task^13,15,30^. The question of whether spatial memory consolidation levels off at some point in time therefore still needs further experimental evaluation.

Our retrospective study design spanned a period of time that exceeds previous prospective and retrospective longitudinal spatial memory experiments. The variance in time since the last zoo visit facilitated a detailed analysis of the time-dependency of performance in all three memory tasks. We observed that the time course of all studied spatial memory representations was nonlinear with a loss of accuracy especially during the initial 3 years. However, performance continued to change at longer memory delays, suggesting that spatial representations continued to transform even at delays that are otherwise considered to be a domain of autobiographical memory testing. The resulting nonlinear forgetting curve for all spatial representations in our study resembles power functions from classical memory experiments such as the Ebbinghaus experiments^17,18,20^. Although primarily obtained with non-sense verbal material on a timescale of minutes to months, studies suggest the applicability to other memory domains, including visual memory^31,32^. However, a comparison of the hypothetical data for spatial representations calculated by the Ebbinghaus equation with our real data revealed that a steeper memory decay of the real data was observed for all three tasks. Notably, the decline of allocentric spatial memory representations was steeper than in the other two tasks. The observed pattern suggests differences in the transformation dynamics of decontextualized material versus integrated spatial representations derived from prior real-world navigational episodes. This indicates that single time-based decay functions may not fully capture the complexity of real-world spatial memory consolidation and/or forgetting. Instead, the temporal trajectories of spatial memory representations over extended periods may be more consistent with frameworks emphasizing distinct memory phases and processes^19^.

The observed differences in the time-dependency of spatial memory performance across tasks further suggests that memories of initial navigational episodes are not stored as holistic representations that consolidate or deteriorate in an all-or-none mode. Instead, increasing memory delay—along with task demands and prior schematic knowledge—may lead to a transformative shift in the respective contributions of different spatial representations to overall spatial memory performance. One factor that critically determines the slope of memory retention across time is intermittent retrieval^20^. Egocentric representations, as assessed with our video-assisted pointing task, are thus in a privileged position, as they recreate the initial navigational experience from a first-person perspective rather than an abstracted allocentric map. Studies have repeatedly shown that egocentric perspective has a beneficial effect on subsequent memory performance^33–35^. Spontaneous intermittent retrieval of a navigational episode between last visit and testing may thus have occurred from a first-person perspective and may have affected the slope of memory retention of egocentric representations but not or much lesser of allocentric representations. Egocentric spatial representations may thus be conceived as the main carriers of episodic spatial information following a navigational experience. In line with this hypothesis, our analyses suggest that the average performance of zoo visitors—particularly in the egocentric task—may not reach the mean performance of zoo-naive controls even in the decades following the observed time span. Although this projection is based on extrapolated data and should be interpreted with caution, it aligns with the notion of a more gradual memory decay for egocentric spatial representations. Conversely, although allocentric representations, as assessed by the target placement task, also relate to initial navigational episodes, their lack of detail and birds-eye perspective render allocentric representations more to gist-like summaries of the spatial layout of the navigational episodes rather than to representations that enable intermittent retrieval of navigational experiences. They may thus be conceived as abstracted templates that may ultimately contribute to an overall spatial ‘zoo’-schema that is independent of a distinct navigational episode^5^. This functional distinction between egocentric and allocentric representations is further supported by the egocentric-to-allocentric task, which captures their interaction and reveals that allocentric memory can potentially compensate for reduced egocentric information and vice versa.

The behaviour of zoo-naive control subjects indeed shows that performance was not solely driven by memory of navigational episodes in the Berlin Zoo but also by a schematic representation independent from the zoo. Thus, visual stimuli may have guided behaviour by prompting the retrieval of schematic representations of previously visited environments that share spatial features with the Berlin Zoo^5^. These schematic representations were particularly effective in the first-person perspective, which aligns with how we typically experience space in everyday life. Supporting this notion, we found that zoo-naive control subjects performed above chance level in all three tasks, with the strongest effects observed in the egocentric and ego- to allocentric task. These findings therefore suggest that spatial memories of navigational episodes acquired in real-world settings are not purely episodic in nature. Instead, they emerge as a combination of the actual navigational experience and semantic knowledge that facilitates logical inferences and decision-making in environments with overlapping spatial features. In this framework, cognitive maps, spatial gist and spatial schema are not three successive steps of abstraction of an initial experience but rather three classes of spatial representations with distinct relationships to navigational episodes that exist in parallel and mutually influence each other^5^.

It should, however, be pointed out that time since last visit explained only up to a quarter of the variance in memory performance across the three tasks. Instead, a combination of factors such as time since last visit, age at last visit and spatial orientation skills can explain up to two thirds of the variance in memory performance. Time thus appears as one of many factors that determines the interplay of spatial representations. This finding aligns with the notion that spatial navigation is influenced by several factors such as age and individual spatial abilities^11,12,36–38^. A further limitation is the variance arising from the ecological setting and uncontrolled confounding factors associated with the retrospective design, which may mask additional temporal differences. In light of this, we caution against overinterpreting the differences in temporal trajectories across tasks in the current study, as they may also reflect task-specific differences in sensitivity or schema availability. Thus, although our data point towards differential memory decay particularly during the first years after acquisition, these patterns probably also reflect memory-independent task-related factors.

Taken together, our findings suggest that memory of navigational episodes emerges from the interaction of multiple parallel spatial representations, each supported by a widespread hippocampal–neocortical network^2,5,38^. The underlying neural substrates are probably dynamically activated to varying degrees based on past experiences and current task demands, with each activation influencing the organization and structure of the retrieved memory^15^. We assume that this process continuously reshapes memory of navigational episodes, reflecting the brain’s ability to adapt and restructure memory networks in response to changing cognitive demands and environmental contexts. This process can be described most appropriately as a memory reorganization process, as it seems likely that the consolidation and forgetting of spatial memories never ends^6,8,9,39^. Combining the retrieval of remote navigational episodes with fMRI measurements may ultimately reveal the underlying neural network dynamics.

## Methods

### Participants

A total of 134 participants were tested (104 zoo visitors, 30 zoo-naive controls; 102 female and 32 male). All participants were recruited via online advertisement and on-site recruitment at the Charité-Universitätsmedizin Berlin. The first recruitment phase took place in August and September 2020 (58 participants) and the second recruitment phase from February 2022 to May 2023 (76 participants). Participants were between 14 and 71 years old, fluent in German, had normal or corrected-to-normal vision, normal hearing, reported being in good health and denied neuropsychiatric disorders or substance abuse. Participants were divided into two groups depending on whether they had visited the zoo at least once (zoo visitors versus zoo-naive controls) regardless of age or other demographic factors. The number of zoo visits varied as well as the age at the last zoo visit (Table 1). No statistical methods were used to predetermine sample sizes, but our sample sizes are comparable to or larger than those reported in previous studies on long-term memory consolidation^23,24^. All participants gave written informed consent. All experimental procedures were conducted in accordance with the Declaration of Helsinki and were approved by the local ethics committee of Charité-Universitätsmedizin Berlin. Participants received €10 per hour as financial compensation. The study was not preregistered.

### Behavioural assessment

#### The Berlin Zoo task

Participants were tested on their spatial memory of the Tierpark Berlin which is one of two zoos in Berlin, Germany. The zoo is a 160-hectare site (Fig. 1) and was founded in 1955. It contains several distinctive landmarks such as a castle, animal enclosures, zoo houses or restaurants that are easily distinguishable from each other.

The participants’ spatial memory of the zoo was tested with three different tasks assessing egocentric and allocentric spatial memory representations (Fig. 2). In addition, participants completed questionnaires to determine covariates (see below). Individual data points per participant were excluded if a landmark that did not exist at the time of the last visit to the zoo was included in the experiment, for example, the monkey house was only opened in 2000.

#### Experimental setup

For memory-guided pointing tasks from a first-person perspective, a total of nine 360° videos (ultra-high resolution, down-sampled from 8K to 4K) were recorded at the zoo in October 2019. The 360° videos were recorded at different locations without overlapping features but at least one distinct environmental landmark (Figs. 1 and 2a). The task was developed in Unity3D (version 2018.2.14f, Unity Technologies) and presented using an HTC VIVE Pro Eye VR headset, provided by a mobile VR laboratory, to fully immerse participants in the zoo environment^40^. The 360°-videos were mapped to the correct video locations using Global Positioning System coordinates and landmarks on a technical map of the zoo embedded in a virtual environment. In addition, the centre of each target position was marked on the technical map. The technical map accurately reflected the spatial structure of the zoo and was provided by Tierpark Berlin authorities for research purposes.

The 2D map used for tasks from a bird’s-eye view were programmed in MATLAB (Mathworks, R2018b) and presented with Psychtoolbox^41^. The task was presented on a Lenovo Thinkpad X1 Carbon laptop (14.0-inch screen). The unlabelled map used was extracted from Google Maps in January 2020 (map data: 2020, Geo-Basis DE/BKG (2009), Google).

#### Experimental procedure

Before testing, all participants were trained for the general design of the pointing tasks. Using a VR headset, they were immersed in a 360° environment close to Brandenburg Gate—a well-known Berlin landmark—where they had to localize themselves and then point in the direction of several remote Berlin landmarks and to the north (Supplementary Information Section 1.1). Then, we assessed the accuracy of memories of navigational episodes in the zoo from a first-person perspective. For this purpose, participants were placed in immersive 360° videos of the zoo. Participants first had to localize themselves within the zoo and were then requested to point precisely to several different remote locations of the zoo (Fig. 2a,b and Supplementary Information Sections 1.2 and 1.3) first using the direction of another zoo location (ego- to allocentric), and second, using the video showing the surroundings (egocentric). After completion of the pointing tasks, we assessed the accuracy of memories of navigational episodes in the zoo from a bird’s-eye view by placing target locations on a 2D map (allocentric). The order of the tasks was fixed to minimize the risk of transfer of spatial knowledge between tasks (Supplementary Information Section 1.4).

Self-localization: participants were asked to report on their location within the zoo verbally (Fig. 2a). The answer was rated as either correct or incorrect. If the answer was incorrect or the participants could not localize themselves at all, they were told the correct answer to avoid errors in the subsequent pointing tasks. A total of nine video locations were tested (Fig. 1, Supplementary Information Section 1.2 and Supplementary Table 2). To ensure that the locations were recognizable, all locations contained at least one specific environmental landmark, such as the terrace in front of Cafe Kakadu (Fig. 2a). Each self-localization was followed by two pointing tasks (Fig. 2b, Supplementary Information Sections 1.3 and 1.4 and Supplementary Table 3).

Direction pointing with video (egocentric): in this pointing task, participants were immersed in the 360°-video and asked to point in the direction of several specific target locations of the zoo, for which they had to relate knowledge of their own position to the features of their immediate environment using the 360°-video. The pointing task always began with pointing to the entrances of the zoo, because each participants’ navigational episodes must have started by entering the zoo via one of these locations. In addition, they were asked to point to two other locations of the zoo, thus yielding a total of three to four location trials per video location (Supplementary Information Section 1.3). Target locations were always separated by at least 45° of visual angle to induce orienting movements of participants. These additional target locations appeared only once during the task and were not repeated at subsequent video positions. In addition, all participants were asked to point to the north at the end of each pointing task to test their global spatial orientation. After completion of the pointing task in the 360° video environment, the next video location was shown and participants were again asked to localize themselves, followed by the next pointing task. The order of the video locations was randomized, but the target locations and their order were kept constant for each video location (Supplementary Information Section 1.3). A total of 20 target locations were tested (Fig. 1).

For the analysis of pointing performance, the angular deviation from the target location was standardized and compared with the *z* score per query of the zoo-naive controls. This procedure was chosen to account for logical inferences about possible target locations in zoo-naive participants, for example, signs or alleys guiding to landmarks.

Direction pointing with arrow (egocentric to allocentric): in this pointing task, the 360° video was removed, and the participants found themselves in a neutral virtual environment with a white floor and a blue sky with several clouds. No landmarks for spatial orientation were provided. They were now asked to imagine that they were standing in the place they had immersed themselves in during self-localization. As an orientation aid, a green circle with a green arrow was projected onto the white floor, pointing in the direction of a second location, for example, the monkey house (Fig. 2b and Supplementary Information Section 1.3). The participants were asked to turn in the direction of the arrow, followed by pointing in the direction of several distinct locations (same locations and order as in direction-pointing with video). This task required the participants to integrate knowledge of their own position and the position of two locations into a global map and to relate these locations to each other spatially. After the completion of the pointing task in the neutral environment, the environment faded out and the original video was shown again (Supplementary Information Section 1.4).

For analysis of pointing performance, the angular deviation from the target location was standardized and compared with the *z*-score per query of the zoo-naive controls as in the pointing task with video. Owing to constraints related to coronavirus disease 2019 during the first data collection phase (August–September 2020), the egocentric-to-allocentric arrow-pointing task could not be administered. This task was added in the second phase of data collection and was completed by all participants recruited during that period (76 participants).

Target placement (allocentric): in this task, participants were asked to place target locations on a 2D map of the zoo from a bird’s-eye view (Fig. 2b). This target placement task requires allocentric representations of the zoo. To place the target locations, participants first saw an isolated image of the location, followed by a fixation cross for 1 s. Then, the map appeared, and the specific location had to be placed on the map by moving the mouse and confirming with a mouse click. Participants had 20 s to decide where to place the specific location and were explicitly asked to place the target locations as precisely as possible even if they could not remember the exact location. After the decision, a fixation cross was displayed again for 1 s and the next trial started. The same 20 target locations were tested as for the pointing tasks (Fig. 1 and Supplementary Information Section 1.3).

For the analysis of placement performance, the raw error distance between the chosen location and the target location was measured in pixels. As possible error distances differ for target locations close to the centre and target locations close to the border of the map, we normalized the error distances to a comparable response range across locations using an accuracy score^42,43^. The accuracy score was calculated as the percentile rank of the raw distance error relative to a distribution of 10,000 possible errors, given the correct location and the spatial boundaries of the map. A score of 1 indicates a perfectly placed location, whereas a score of 0 reflects the worst possible placed location. The accuracy score was standardized and compared with the trial-wise *z*-scores of the zoo-naive controls. For comparison across tasks the *z*-score was multiplied by −1. Trials in which objects were placed outside the unlabelled map were excluded from the analysis.

### Assessment of covariates

All participants completed socio-demographic questionnaires. These included age, biological sex, years of education and handedness. In addition, all zoo visitors stated how often they had visited the zoo in total and provided the approximate dates of their first and last visits. The age at the last zoo visit was calculated using the age at testing and the date of the last zoo visit (Table 1).

To assess spatial abilities, all participants completed a German version of the SBSOD^44^. Responses were rated on a Likert scale from 1 to 7. For the SBSODs, a higher final score indicated a better sense of direction.

### Statistical analysis

Statistical analyses were performed in RStudio (v. 4.4.1). To compare the responses of the zoo visitors with the performance of the control participants and to account for the different localization properties in the three tasks, we first converted the raw scores of all participants into *z*-scores. Our primary goal was to achieve comparability across tasks measured on different scales (deviation in degrees for the pointing tasks and a distance-based accuracy score for target placement) rather than to enforce normality of the raw score distributions. For this purpose, we applied *z*-score standardization as a linear transformation. These *z*-scores are based on the average performance of the control participants for each query in the pointing task and for each target placement in the target placement task. We then aggregated the data per participant in each task.

Next, we visually analysed the *z*-scores of the three tasks and examined skewness and kurtosis of the aggregated data. Then, we used the Shapiro–Wilk test to formally check whether the data of the three tasks were normally distributed. As the assumption of a normal distribution had to be rejected for most variables, we compared the performance of zoo visitors and the zoo-naive controls with a non-parametric one-sided Mann–Whitney *U* test (Fig. 3 and Supplementary Information Section 1.6). The comparison between the three experimental tasks was performed using a Kruskal–Wallis test. Post hoc tests were conducted using pairwise Mann–Whitney *U* tests. *P* values were corrected for multiple comparisons using the Bonferroni method. We used a permutation test with 10,000 permutations to assess whether the observed group-level error in performance of the zoo-naive controls differed from chance. For effect sizes, *r* was calculated for the Mann–Whitney *U* test and the permutation test using the formula *r* = *Z*/√(*n*). *Z* was derived by the quantile of the *P* value (small, 0.1 ≤ *r* < 0.3; medium, 0.3 ≤ *r* < 0.5; large, *r* ≥ 0.5). Cohen’s *d* was calculated for bootstrapping, calculated as the mean difference between groups divided by the pooled standard deviation (small, *d* ≥ 0.2; medium, *d* ≥ 0.5; large, *d* ≥ 0.8). *η*^2^ was calculated for the Kruskal–Wallis test with the rcompanion R package as *η*^2^ = (*H* − *k* + 1)/(*n* − *k*) (small, 0.01 ≤ *η*^2^ < 0.06; medium, 0.06 ≤ *η*^2^ < 0.14; large, *η*^2^ ≥ 0.14).

Correlations between task performance and covariates were calculated using Spearman’s rank sum correlation. Comparison of the correlation-coefficients between tasks was conducted with a Fisher’s *z*-test.

To uncover the relationship between time and spatial memory performance, we first used eight different regression models to investigate which regression would best explain the collected data across the investigated memory delays (Fig. 4 and Supplementary Information Section 1.7). For logarithmic, exponential and power regressions, the respective data were log-transformed after being converted to a positive value. The lowest *z*-scores across conditions were approximately −1.5. To ensure all values were positive and to allow valid comparisons across conditions, we added a constant of +2 to all *z*-scores before applying the logarithmic transformation. This procedure preserved the relative differences between values while enabling statistical analysis. Second, we selected two time intervals with at least 20 observations for each task, which were aggregated into bins to compare performance between zoo visitors and zoo-naive controls, as well as across tasks, for memory delays beyond the first 356 days. The intervals 1–10 years and 10–30 years met these criteria and were compared using non-parametric tests (Fig 5b).

Owing to a sample size of *n* = 104, we performed regression validation with leave-one-out cross-validation. The Akaike information criterion was chosen for model selection as it is also valid for nonlinear regressions. Further model descriptors were the residual standard error, the mean square error, the root mean square error, *R*^2^ (adj.) and the Bayesian information criterion (Supplementary Information Section 1.6). A two-way analysis of variance was performed for each regression analysis to examine whether spatial memory performance changed over time across the tasks. Post hoc pairwise comparisons of slopes were performed, and *P* values were corrected for multiple comparisons using the Tukey method. Effect sizes were assessed using *ꞷ*^2^, calculated with the effect size R package as *ꞷ*^2^ = (SS_effect _− df_effect _× MS_error_)/(SS_total _+ MS_error_), where SS denotes the sum of squares, df the degrees of freedom and MS the mean square error (small, 0.01 ≤ *ꞷ*^2^ < 0.06; medium, 0.06 ≤ *ꞷ*^2^ < 0.14; large, *ꞷ*^2^ ≥ 0.14). Moreover, task-specific differences over time were evaluated by computing estimated marginal means from a GAM on a 6–10,000-day prediction grid and calculating pairwise contrasts between tasks at each time point.

To compare the observed memory decay with a classical and highly influential forgetting model, we generated simulated decay trajectories using a modified Ebbinghaus forgetting function: −1.84/(log_10_(time)^1.25^ + 1.84) (refs. ^17,18^). The function was evaluated across the observed time range from 6 days to 30 years, converted to minutes for the respective calculations. Each task’s simulated curve was scaled to match the modelled starting value from the respective power regression at minute 1 (direction-pointing (video), −1.042; direction-pointing (arrow), −1.321; target placement, −1.578). We used a linear model on log-transformed data points to assess differences in decay trajectories between simulated and real data across tasks. Estimated marginal means were calculated, and planned contrasts were conducted to compare real and simulated data for each task. Additional post hoc pairwise contrasts tested for differences between decay trajectories within and across tasks.

To investigate the contribution of multiple factors to spatial memory performance, we applied a GAM. In addition to the time since last visit, we examined other zoo-visit-related factors, including the time since the first visit, the total number of zoo visits and age at the last visit. Personal characteristics such as age and educational level were also considered. In addition, spatial strategies and abilities, such as northward orientation that is the ability to point to the north, were included in the model. The final model selected was the simplest model that best explained spatial memory consolidation, incorporating only the most relevant and significant contributing factors (Table 2).

The level of significance was set to the conventional level of *P* < 0.05.

### Reporting summary

Further information on research design is available in the Nature Portfolio Reporting Summary linked to this article.

## Supplementary information

Supplementary Information Supplementary Information Sections 1.1–1.8, containing Supplementary Tables 1–5, Supplementary Figs. 1 and 2.

Reporting summary

## Data Availability

The data and R code of this study are available via OSF at https://osf.io/sb65k/.

## Code availability

The R and MATLAB code of this study are available via OSF at https://osf.io/sb65k/.
