# Supplementary material for: Long-term memory reorganization of navigational episodes
Source: Nat Hum Behav. 2026 May 18;10(7):1327–39. doi: 10.1038/s41562-026-02472-x (PMC13388105; doi:10.1038/s41562-026-02472-x)
Supplement: Supplementary file 1 — Supplementary Information Sections 1.1–1.8, containing Supplementary Tables 1–5, Supplementary Figs. 1 and 2. [file 41562_2026_2472_MOESM1_ESM.pdf]

nature human behaviour

Supplementary information https://doi.org/10.1038/s41562-026-02472-x

Long-term memory reorganization of navigational episodes

In the format provided by the authors and unedited

Supplementary material

1.1

Table 1. Descriptive location data for training task. Pointing from video location to target location.

Trial	Video location	Target location	Euclidean distance	Video to target location angle	Auxiliary arrow points to
**Direction of target locations (ego- to allocentric)**					
1	Pariser Platz	Brandenburg Gate	68m	-89.6°	TV Tower
2	Pariser Platz	Parliament	886m	-38.6°	TV Tower
3	Pariser Platz	North	n.a.	0°	TV Tower
**Direction of target locations (egocentric)**					
1	Pariser Platz	Brandenburg Gate	702m	-89.6°	n.a.
2	Pariser Platz	Parliament	886m	-38.6°	n.a.
3	Pariser Platz	North	n.a.	0°	n.a.

1.2

Table 2. Descriptive target location data. Data presented as percentage of correct responses. Data on self-localization only available for video locations (Note: n.a. = not applicable.

Location no.	Location name	Self-Localization (correct first response in %)
1	Giant tortoise house	n.a.
2	Castle Friedrichsfelde	90%
3	Monkey house	17%
4	Restaurant Patagona	65%
5	Rock kangaroos	n.a.
6	Lemur woods	n.a.
7	Giraffe house	100%
8	Flamingo lagoon	n.a.
9	Dromedar-/ Camel pasture	n.a.

10	Pachyderm house	99%
11	Grevy-Zebra	n.a.
12	Water playground	n.a,
13	Kieke-Mal-Waldbühne (bird show)	n.a.
14	Predator house	79%
15	African penguin	n.a.
16	Entrance (Bear)	94%
17	Terrace café Kakadu	95%
18	Petting zoo	n.a.
19	Polar bear	70%
20	Spectacled bear	n.a.

1.3

Table 3. Descriptive location data. Pointing from video location to target location. Letters and numbers refer to the overview map provided in figure 1. The order of video locations was randomized, the trial order was fixed.

Trial	Video location	Target location	Euclidean distance	Video to target location angle	Auxiliary arrow points to
	**1. Direction of target locations (ego- to allocentric)**				
1	A Castle Friedrichsfelde	16 Entrance (Bear)	702m	176.6°	Predator house
2	A Castle Friedrichsfelde	11 Grevy-Zebra	886m	115.5°	Predator house
3	A Castle Friedrichsfelde	17 Terrace café Kakadu	692m	158.3°	Predator house
4	A Castle Friedrichsfelde	North	n.a.	0.0°	Predator house
	**2. Direction of target locations (egocentric)**				
1	A Castle Friedrichsfelde	16 Entrance (Bear)	702m	176.6°	n.a.
2	A Castle Friedrichsfelde	11 Grevy-Zebra	886m	115.5°	n.a.
3	A Castle Friedrichsfelde	17 Terrace café Kakadu	692m	158.3°	n.a.
4	A Castle Friedrichsfelde	North	n.a.	0.0°	n.a.

	**1. Direction of target locations (ego- to allocentric)**				
1	B Monkey house	2 Entry (Castle)	659m	- 121.5°	Restaurant Patagona
2	B Monkey house	16 Entry (Bear)	920m	- 153.1°	Restaurant Patagona
3	B Monkey house	7 Giraffe house	508m	155.3°	Restaurant Patagona
4	B Monkey house	6 Lemur woods	540m	174.3°	Restaurant Patagona
5	B Monkey house	North	n.a.	0.0°	Restaurant Patagona
	**2. Direction of target locations (egocentric)**				
1	B Monkey house	2 Entry (Castle)	659m	- 121.5°	n.a.
2	B Monkey house	16 Entry (Bear)	920m	- 153.1°	n.a.
3	B Monkey house	7 Giraffe house	508m	155.3°	n.a.
4	B Monkey house	6 Lemur woods	540m	174.3°	n.a.
5	B Monkey house	North	n.a.	0.0°	n.a.
	**1. Direction of target locations (ego- to allocentric)**				
1	C Restaurant Patagona	16 Entry (Bear)	814m	- 150.4°	Giraffe house
2	C Restaurant Patagona	2 Entry (Castle)	364m	- 86.5°	Giraffe house
3	C Restaurant Patagona	12 Water playground	571m	173.7°	Giraffe house
4	C Restaurant Patagona	3 Monkey house	390m	25.1°	Giraffe house
5	C Restaurant Patagona	North	n.a.	0.0°	Giraffe house
	**2. Direction of target locations (egocentric)**				
1	C Restaurant Patagona	16 Entry (Bear)	814m	- 150.4°	n.a.
2	C Restaurant Patagona	2 Entry (Castle)	364m	- 86.5°	n.a.
3	C Restaurant Patagona	12 Water playground	571m	173.7°	n.a.
4	C Restaurant Patagona	3 Monkey house	390m	25.1°	n.a.
5	C Restaurant Patagona	North	n.a.	0.0°	n.a.
	**1. Direction of target locations (ego- to allocentric)**				
1	D Giraffe house	16 Entry (Bear)	920m	- 131.1°	Terrace café Kakadu
2	D Giraffe house	2 Entry (Castle)	711m	- 85.8°	Terrace café Kakadu

3	D Giraffe house	9 Dromedar-/ Camel pasture	427m	- 129.5°	Terrace café Kakadu
4	D Giraffe house	5 Rock kangaroos	281m	- 91.0°	Terrace café Kakadu
5	D Giraffe house	North	n.a.	0.0°	Terrace café Kakadu
	**2. Direction of target locations (egocentric)**				
1	D Giraffe house	16 Entry (Bear)	920m	- 131.1°	n.a.
2	D Giraffe house	2 Entry (Castle)	711m	- 85.8°	n.a.
3	D Giraffe house	9 Dromedar-/ Camel pasture	427m	- 129.5°	n.a.
4	D Giraffe house	5 Rock kangaroos	281m	- 91.0°	n.a.
5	D Giraffe house	North	n.a.	0.0°	n.a.
	**1. Direction of target locations (ego- to allocentric)**				
1	E Pachyderm house	2 Entry (Castle)	716m	- 71.9	Monkey house
2	E Pachyderm house	16 Entry (Bear)	751m	- 117.2°	Monkey house
3	E Pachyderm house	15 African penguin	327m	162.1°	Monkey house
4	E Pachyderm house	19 Polar bear	646m	- 137.0	Monkey house
5	E Pachyderm house	North	n.a.	0.0°	Monkey house
	**2. Direction of target locations (egocentric)**				
1	E Pachyderm house	2 Entry (Castle)	716m	- 71.9	n.a.
2	E Pachyderm house	16 Entry (Bear)	751m	- 117.2°	n.a.
3	E Pachyderm house	15 African penguin	327m	162.1°	n.a.
4	E Pachyderm house	19 Polar bear	646m	- 137.0	n.a.
5	E Pachyderm house	North	n.a.	0.0°	n.a.
	**1. Direction of target locations (ego- to allocentric)**				
1	F Predator house	2 Entry (Castle)	915m	- 60.9°	Polar bear
2	F Predator house	16 Entry (Bear)	746m	- 107.0°	Polar bear
3	F Predator house	10 Pachyderm house	251m	- 28.7°	Polar bear
4	F Predator house	18 Petting Zoo	207m	- 127.2°	Polar bear
5	F Predator house	North	n.a.	0.0°	Polar bear

	**2. Direction of target locations (egocentric)**				
1	F Predator house	2 Entry (Castle)	915m	- 60.9°	n.a.
2	F Predator house	16 Entry (Bear)	746m	- 107.0°	n.a.
3	F Predator house	10 Pachyderm house	251m	- 28.7°	n.a.
4	F Predator house	18 Petting Zoo	207m	- 127.2°	n.a.
5	F Predator house	North	n.a.	0.0°	n.a.
	**1. Direction of target locations (ego- to allocentric)**				
1	G Terrace café Kakadu	16 Entry (Bear)	221m	- 113.9°	Monkey house
2	G Terrace café Kakadu	2 Entry (Castle)	692m	- 23.6°	Monkey house
3	G Terrace café Kakadu	20 Spectacled bear	216m	- 177.1°	Monkey house
4	G Terrace café Kakadu	13 Kieke-Mal-Waldbühne (bird show)	245m	75.2°	Monkey house
5	G Terrace café Kakadu	North	n.a.	0.0°	Monkey house
	**2. Direction of target locations (egocentric)**				
1	G Terrace café Kakadu	16 Entry (Bear)	221m	- 113.9°	n.a.
2	G Terrace café Kakadu	2 Entry (Castle)	692m	- 23.6°	n.a.
3	G Terrace café Kakadu	20 Spectacled bear	216m	- 177.1°	n.a.
4	G Terrace café Kakadu	13 Kieke-Mal-Waldbühne (bird show)	245m	75.2°	n.a.
5	G Terrace café Kakadu	North	n.a.	0.0°	n.a.
	**1. Direction of target locations (ego- to allocentric)**				
1	H Entry (Bear)	2 Entry (Castle)	702m	- 5.8°	Polar bear
2	H Entry (Bear)	8 Flamingo lagoon	601m	40.7°	Polar bear
3	H Entry (Bear)	1 Giant tortoise	713m	-9.5°	Polar bear
4	H Entry (Bear)	North	n.a.	0.0°	Polar bear
	**2. Direction of target locations (egocentric)**				
1	H Entry (Bear)	2 Entry (Castle)	702m	- 5.8°	n.a.
2	H Entry (Bear)	8 Flamingo lagoon	601m	40.7°	n.a.

3	H Entry (Bear)	1 Giant tortoise	713m	-9.5°	n.a.
4	H Entry (Bear)	North	n.a.	0.0°	n.a.
**1. Direction of target locations (ego- to allocentric)**					
1	I Polar bear	16 Entry (Bear)	304m	- 66.4	Pachyderm house
2	I Polar bear	2 Entry (Castle)	885m	- 21.7°	Pachyderm house
3	I Polar bear	14 Predator house	444m	62.6°	Pachyderm house
4	I Polar bear	4 Restaurant Patagona	874m	8.1°	Pachyderm house
5	I Polar bear	North	n.a.	0.0°	Pachyderm house
**2. Direction of target locations (egocentric)**					
1	I Polar bear	16 Entry (Bear)	304m	- 66.4	n.a.
2	I Polar bear	2 Entry (Castle)	885m	- 21.7°	n.a.
3	I Polar bear	14 Predator house	444m	62.6°	n.a.
4	I Polar bear	4 Restaurant Patagona	874m	8.1°	n.a.
5	I Polar bear	North	n.a.	0.0°	n.a.

1.4

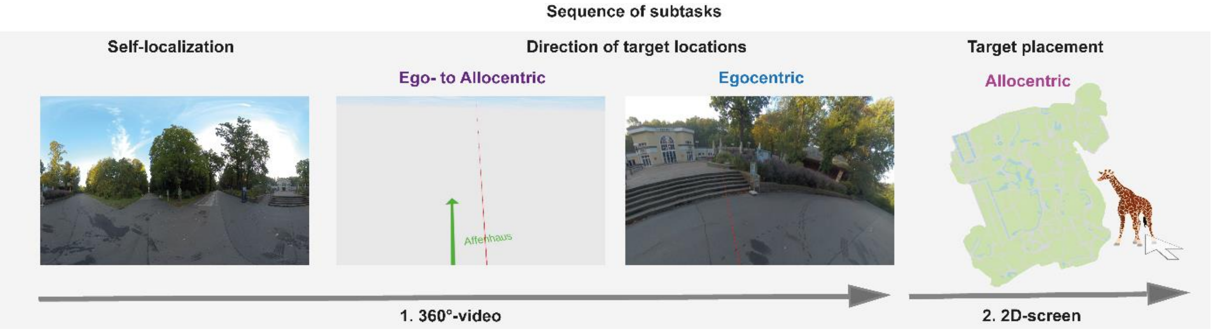

Figure 1. The order of the tasks in the Berlin Zoo task. To avoid a transfer of spatial knowledge between the subtasks, self-localization was followed by the pointing task with arrow, followed by the pointing task with video until all video locations and their respective target locations were tested and finally the target placement. Map data © OpenStreetMap contributors (www.openstreetmap.org/copyright).

1.5

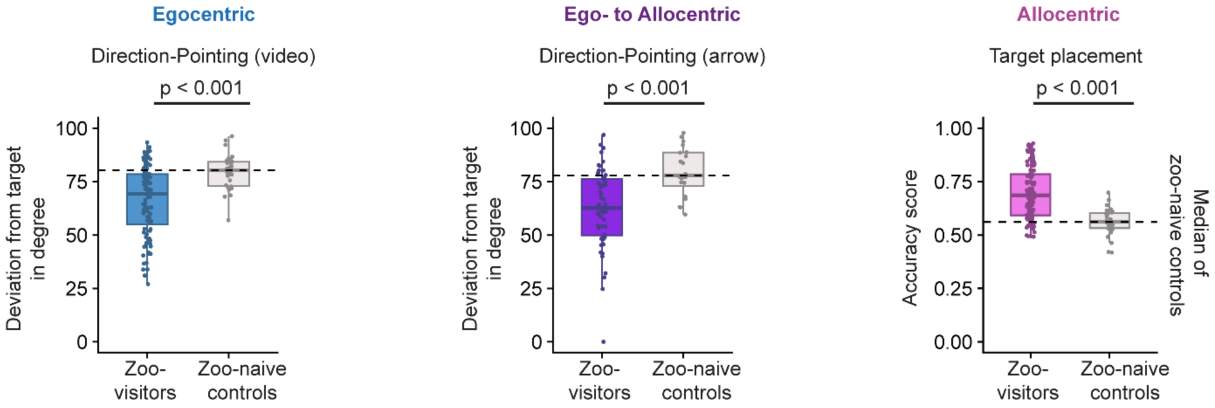

Figure 2. Zoo visitors showed a lower deviation from target locations as well as a higher accuracy score and outperformed zoo-naïve controls in all three tasks. Data presented as boxplots with a centre line as median, Tukey-style whiskers extend 1.5 times the interquartile range from 25th and 75th percentiles and dots represent individual datapoints. Comparison was conducted using a Mann-Whitney U Test. Zoo-visitors: n = 104 for direction-pointing (video) & target placement, n = 56 for direction-pointing (arrow); zoo-naïve controls: n = 30 for direction-pointing (video) & target placement, n = 20 for direction-pointing (arrow).

1.6

Table 4. Table of results for the comparison of memory accuracy as z-score between zoo visitors and zoo-naive control per subtask. Data presented as median and IQR. Group-comparison with Mann-Whitney U-Test.

	**Zoo visitors**	**Zoo-naïve control**	**p-value**	**95% CI**
**Self-localization (%)**	77 (67 – 89)	77 (67 – 77)	0.013	3.416^{-5}, inf
**Direction-Pointing (video)**	-0.226 (-0.498 - -0.036)	-0.016 (-0.143 - 0.138)	< 0.001	-inf, -0.152
**Direction-Pointing (arrow)**	-0.310 (-0.576 - -0.048)	-0.021 (-0.134 - 0.169)	< 0.001	-inf, -0.168
**Target placement**	0.455 (-0.149 - 0.843)	0.018 (-0.114 – 0.126)	< 0.001	-inf, -0.467

1.7

Table 5. Table of results. Correlation and functions explaining the variance in spatial memory performance by elapsed time since last visit.

	**Direction-pointing (video) a**	**Direction-pointing (arrow) b**	**Target placement c**	**Test statistic**
**Spearman's correlation**	Rho(102) = 0.25,<br>p = 0.010	Rho(54) = 0.370,<br>p = 0.004	Rho(102) = 0.35,<br>p < 0.001	a vs. b: z = -1.521, p = 0.128<br>a vs. c: z = -1.038, p = 0.299<br>b vs. c: z = -0.346, p = 0.729
**Linear regression**	R^2_{adj} = 0.058<br>$F_{(1,100)}$ = 7.299<br>p = 0.008<br>RSE = 0.329	R^2_{adj} = 0.091<br>$F_{(1,53)}$ = 6.519<br>p = 0.014<br>RSE = 0.342	R^2_{adj} = 0.159<br>$F_{(1,100)}$ = 20.299<br>p < 0.001<br>RSE = 0.392	Task:<br>$F_{(2,256)}$ = 10.429, p < 0.001<br>Time since last visit:<br>$F_{(1,256)}$ = 33.463, p < 0.001

	MSE = 0.113<br>RMSE = 0.336<br>AIC = 69.046<br>BIC = 76.951	MSE = 0.126<br>RMSE = 0.354<br>AIC = 44.727<br>BIC = 50.803	MSE = 0.160<br>RMSE = 0.40<br>AIC = 105.448<br>BIC = 113.352	Task*elapsed time:<br>$F_{(2,256)} = 1.518$, $p = 0.221$
**Logarithmic regression**<br>(log-transformation of the predictor variable)	$R^2_{adj} = 0.122$<br>$F_{(1,100)} = 15.166$<br>$p < 0.001$<br>RSE = 0.317<br>MSE = 0.104<br>RMSE = 0.323<br>AIC = 61.823<br>BIC = 69.727	$R^2_{adj} = 0.201$<br>$F_{(1,53)} = 14.838$<br>$p < 0.001$<br>RSE = 0.321<br>MSE = 0.110<br>RMSE = 0.331<br>AIC = 37.515<br>BIC = 43.591	$R^2_{adj} = 0.230$<br>$F_{(1,100)} = 31.418$<br>$p < 0.001$<br>RSE = 0.375<br>MSE = 0.146<br>RMSE = 0.382<br>AIC = 96.414<br>BIC = 104.319	Task:<br>$F_{(2,256)} = 11.407$, $p < 0.001$<br>Time since last visit:<br>$F_{(1,256)} = 60.703$, $p < 0.001$<br>Task*elapsed time:<br>$F_{(2,256)} = 1.620$, $p = 0.199$
**Exponential regression**<br>(log-transformation of the dependent variable)	$R^2_{adj} = 0.050$<br>$F_{(1,100)} = 6.323$<br>$p = 0.014$<br>RSE = 0.331<br>MSE = 0.116<br>RMSE = 0.340<br>AIC = -20.141<br>BIC = -12.237	$R^2_{adj} = 0.096$<br>$F_{(1,53)} = 6.837$<br>$p = 0.011$<br>RSE = 0.345<br>MSE = 0.128<br>RMSE = 0.358<br>AIC = -9.011<br>BIC = -2.935	$R^2_{adj} = 0.147$<br>$F_{(1,100)} = 18.619$<br>$p < 0.001$<br>RSE = 0.399<br>MSE = 0.166<br>RMSE = 0.407<br>AIC = 47.297<br>BIC = 55.201	Task:<br>$F_{(2,256)} = 11.957$, $p < 0.001$<br>Time since last visit:<br>$F_{(1,256)} = 31.386$, $p < 0.001$<br>Task*elapsed time:<br>$F_{(2,256)} = 2.256$, $p = 0.107$
**Power regression**<br>(log-transformation for the dependent and predictor variable)	$R^2_{adj} = 0.101$<br>$F_{(1,100)} = 12.466$<br>$p < 0.001$<br>RSE = 0.319<br>MSE = 0.106<br>RMSE = 0.325<br>AIC = -25.875<br>BIC = -17.971	$R^2_{adj} = 0.227$<br>$F_{(1,53)} = 17.139$<br>$p < 0.001$<br>RSE = 0.323<br>MSE = 0.112<br>RMSE = 0.334<br>AIC = -17.772<br>BIC = -11.696	$R^2_{adj} = 0.237$<br>$F_{(1,100)} = 32.680$<br>$p < 0.001$<br>RSE = 0.379<br>MSE = 0.149<br>RMSE = 0.387<br>AIC = 35.850<br>BIC = 43.754	Task:<br>$F_{(2,256)} = 13.235$, $p < 0.001$<br>Time since last visit:<br>$F_{(1,256)} = 60.390$, $p < 0.001$<br>Task*elapsed time:<br>$F_{(2,256)} = 3.355$, **$p = 0.036$**<br>Post-hoc comparison (Tukey):<br>a vs. b: $p = 0.555$<br>a vs. c: **$p = 0.027$**<br>b vs. c: $p = 0.529$
**Quadratic polynomic regression**	$R^2_{adj} = 0.148$<br>$F_{(2,99)} = 9.822$<br>$p < 0.001$<br>RSE = 0.311<br>MSE = 0.104<br>RMSE = 0.322<br>AIC = 59.760<br>BIC = 70.299	$R^2_{adj} = 0.132$<br>$F_{(2,,52)} = 5.188$<br>$p = 0.009$<br>RSE = 0.331<br>MSE = 0.121<br>RMSE = 0.348<br>AIC = 43.097<br>BIC = 51.199	$R^2_{adj} = 0.173$<br>$F_{(2,99)} = 11.70$<br>$p < 0.001$<br>RSE = 0.387<br>MSE = 0.160<br>RMSE = 0.401<br>AIC = 104.654<br>BIC = 115.193	Task:<br>$F_{(2,253)} = 10.957$, $p < 0.001$<br>Time since last visit:<br>$F_{(2,253)} = 25.141$, $p < 0.001$<br>Task*elapsed time:<br>$F_{(4,253)} = 1.008$, $p = 0.407$

**Cubic polynomic regression**	$R^2_{adj} = 0.139$<br>$F_{(3,98)} = 6.492$<br>$p < 0.001$<br>RSE = 0.311<br>MSE = 0.110<br>RMSE = 0.332<br>AIC = 61.736<br>BIC = 74.909	$R^2_{adj} = 0.120$<br>$F_{(3,,51)} = 3.490$<br>$p = 0.022$<br>RSE = 0.330<br>MSE = 0.125<br>RMSE = 0.354<br>AIC = 44.838<br>BIC = 54.965	$R^2_{adj} = 0.197$<br>$F_{(3,98)} = 9.334$<br>$p < 0.001$<br>RSE = 0.379<br>MSE = 0.154<br>RMSE = 0.393<br>AIC = 102.656<br>BIC = 115.830	Task:<br>$F_{(2,250)} = 11.043$, $p < 0.001$<br>Time since last visit:<br>$F_{(3,250)} = 17.714$, $p < 0.001$<br>Task*elapsed time:<br>$F_{(6,250)} = 1.10$, $p = 0.363$
**Spline regression**	$R^2_{adj} = 0.159$<br>$F_{(7,94)} = 4.756$<br>$p = 0.001$<br>RSE = 0.301<br>MSE = 0.104<br>RMSE = 0.323<br>Knots = 180, 2000, 4000, 7500<br>AIC = 63.069<br>BIC = 86.781	$R^2_{adj} = 0.209$<br>$F_{(6,48)} = 3.072$<br>$p = 0.009$<br>RSE = 0.301<br>MSE = 0.119<br>RMSE = 0.344<br>Knots = 180, 1000, 2000, 6500<br>AIC = 42.381<br>BIC = 60.609	$R^2_{adj} = 0.236$<br>$F_{(7,94)} = 5.505$<br>$p < 0.001$<br>RSE = 0.363<br>MSE = 0.155<br>RMSE = 0.394<br>Knots = 180, 1000, 4000, 6000<br>AIC = 101.244<br>BIC = 115.498	Task:<br>$F_{(2,238)} = 11.519$, $p < 0.001$<br>Time since last visit:<br>$F_{(7,238)} = 9.072$, $p < 0.001$<br>Task*elapsed time:<br>$F_{(14,238)} = 1.541$, $p = 0.098$<br>Knots = 180, 2000, 4000, 6000
**Generalized additive model**<br>(local smoothing regression of the predictor variable)	$R^2_{adj} = 0.171$<br>$F_{(2.57)} = 3.572$<br>$p = 0.002$<br>RSE = 0.301<br>MSE = 0.116<br>RMSE = 0.341<br>Deviance expl.: 21.8%<br>GCV = 0.103<br>AIC = 60.517<br>BIC = 81.075	$R^2_{adj} = 0.129$<br>$F_{(2.89)} = 3.796$<br>$p = 0.024$<br>RSE = 0.332<br>MSE = 0.135<br>RMSE = 0.367<br>Deviance expl.: 15.8%<br>GCV = 0.123<br>AIC = 43.227<br>BIC = 51.104	$R^2_{adj} = 0.189$<br>$F_{(3.65)} = 7.576$<br>$p < 0.001$<br>RSE = 0.382<br>MSE = 0.161<br>RMSE = 0.401<br>Deviance expl.: 20.9%<br>GCV = 0.157<br>AIC = 103.333<br>BIC = 115.498	Task:<br>$F_{(2,253)} = 10.88$, $p < 0.001$<br>Time since last visit:<br>$F_{(2,253)} = 8.862$, $p < 0.001$<br>Task*elapsed time:<br>n.a.

In the pointing tasks and the target placement task, the z-score was based on a metric deviation from the target, so that negative correlations were associated with increased memory performance. The blue colour indicates positive correlations in graded weighting. All correlations were Spearman's correlation. Comparison of correlations were conducted with Fishers z-transformation. All models are presented with their key data to explain the variance of spatial memory performance. Note: n.a. = not applicable

1.8

Table 6. Table of best fitting model formulas and calculations spanning three decades.

	Direction-pointing (video)	Direction-pointing (arrow)	Target placement

Mean and upper to lower standard deviation of zoo-naïve controls (Additionally, positively transformed (+2) and logarithmized z-scores).	Mean ± SD: 0.0 ± 0.174<br>log-transformed mean ± SD: 0.693 ± (0.602 - 0.777)	Mean ± SD: 0.0 ± 0.227<br>log-transformed mean ± SD: 0.693 ± (0.573 - 0.801)	Mean ± SD: -0.0 ± 0.229<br>log-transformed mean ± SD: 0.692 ± (0.571 - 0.802)
Best fitting and interpretable model with coefficients	**Power regression**<br>**Intercept:** 0.2479851<br>**Log(days):** 0.04005188<br>Results are given on log-scale not response scale	**Power regression**<br>**Intercept:** 0.07381116<br>**Log(days):** 0.06331582<br>Results are given on log-scale not response scale	**Power regression**<br>**Intercept:** -0.2253<br>**Log(days):** 0.0875<br>Results are given on log-scale not response scale
Timepoint when visitors and controls perform similarly	Mean: 0.2479851 + 0.04005188* log(**67147 days**) = 0.693<br>SD: 0.2479851 + 0.04005188* log(**6932 days**) = 0.602	Mean: 0.07381116 + 0.06331582* log(**17707 days**) = 0.693<br>SD: 0.07381116 + 0.0633158* log (**2653 days**) = 0.573	Mean: -0.2253 + 0.0875* log(**35865 days**) = 0.692<br>SD: -0.2253 + 0.0875* log (**8910 days**) = 0.571
**Timepoint**			
**Quarter year/ (91.3 days)**	0.429 (0.366 - 0.491)	0.36 (0.273 - 0.446)	0.17 (0.085 - 0.254)
**Half year (182.5 days)**	0.457 (0.405 - 0.508)	0.403 (0.332 - 0.475)	0.23 (0.161 - 0.3)
**Year 1 (365 days)**	0.484 (0.44 - 0.528)	0.447 (0.388 - 0.507)	0.291 (0.203 - 0.35)
**Year 2 (730 days)**	0.512 (0.471 - 0.553)	0.491 (0.438 - 0.545)	0.352 (0.296 - 0.407)
**Year 3 (1095 days)**	0.528 (0.486 - 0.57)	0.517 (0.463 - 0.571)	0.387 (0.331 - 0.443)
**Year 4 (1460 days)**	0.54 (0.496 - 0.583)	0.535 (0.479 - 0.591)	0.412 (0.353 - 0.465)
**Year 5 (1825 days)**	0.549 (0.503 - 0.594)	0.549 (0.491 - 0.608)	0.432 (0.37 - 0.493)
**Year 6 (2190 days)**	0.556 (0.508 - 0.604)	0.561 (0.5 - 0.622)	0.448 (0.384 - 0.512)
**Year 7 (2555 days)**	0.562 (0.513 - 0.612)	0.571 (0.507 - 0.634)	0.461 (0.395 - 0.528)
**Year 8 (2920 days)**	0.568 (0.516 - 0.619)	0.579 (0.514 - 0.644)	0.473 (0.404 - 0.542)
**Year 9 (3285 days)**	0.572 (0.519 - 0.625)	0.586 (0.519 - 0.654)	0.483 (0.412 - 0.554)
**Year 10 (3650 days)**	0.577 (0.522 - 0.631)	0.593 (0.524 - 0.663)	0.492 (0.419 - 0.566)
**Year 11 (4015 days)**	0.58 (0.525 - 0.636)	0.599 (0.528 - 0.671)	0.501 (0.426 - 0.576)
**Year 12 (4380 days)**	0.584 (0.527 - 0.641)	0.605 (0.531 - 0.678)	0.508 (0.431 - 0.585)
**Year 13 (4745 days)**	0.587 (0.529 - 0.645)	0.61 (0.535 - 0.685)	0.515 (0.437 - 0.594)
**Year 14 (5110 days)**	0.59 (0.53 - 0.65)	0.614 (0.538 - 0.691)	0.522 (0.441 - 0.602)
**Year 15 (5475 days)**	0.593 (0.532 - 0.653)	0.619 (0.541 - 0.697)	0.528 (0.446 - 0.61)

**Year 16 (5840 days)**	0.595 (0.534 - 0.657)	0.623 (0.543 - 0.702)	0.534 (0.45 - 0.617)
**Year 17 (6205 days)**	0.598 (0.535 - 0.661)	0.627 (0.546 - 0.708)	0.539 (0.454 - 0.624)
**Year 18 (6570 days)**	0.6 (0.536 - 0.664)	0.63 (0.548 - 0.713)	0.544 (0.458 - 0.63)
**Year 19 (6935 days)**	0.602 (0.537 - 0.667)	0.634 (0.55 - 0.717)	0.549 (0.461 - 0.636)
**Year 20 (7300 days)**	0.604 (0.539 - 0.67)	0.637 (0.552 - 0.722)	0.553 (0.464 - 0.64)
**Year 21 (7665 days)**	0.606 (0.54 - 0.673)	0.64 (0.554 - 0.726)	0.557 (0.468 - 0.647)
**Year 22 (8030 days)**	0.608 (0.541 - 0.675)	0.643 (0.556 - 0.73)	0.561 (0.471 - 0.652)
**Year 23 (8395 days)**	0.61 (0.542 - 0.678)	0.646 (0.558 - 0.734)	0.565 (0.473 - 0.657)
**Year 24 (8760 days)**	0.612 (0.543 - 0.68)	0.649 (0.559 - 0.738)	0.57 (0.477 - 0.663)
**Year 25 (9125 days)**	0.613 (0.544 - 0.683)	0.651 (0.561 - 0.741)	0.573 (0.479 - 0.667)
**Year 26 (9490 days)**	0.615 (0.544 - 0.685)	0.654 (0.563 - 0.745)	0.576 (0.481 - 0.671)
**Year 27 (9855 days)**	0.616 (0.545 - 0.687)	0.656 (0.564 - 0.748)	0.579 (0.483 - 0.675)
**Year 28 (10220 days)**	0.618 (0.546 - 0.689)	0.658 (0.565 - 0.751)	0.583 (0.486 - 0.679)
**Year 29 (10585 days)**	0.619 (0.547 - 0.692)	0.661 (0.567 - 0.754)	0.586 (0.488 - 0.682)
**Year 30 (10950 days)**	0.62 (0.547 - 0.693)	0.662 (0.568 - 0.757)	0.588 (0.49 - 0.686)
