# Supplementary material for: Long-term memory reorganization of navigational episodes
Source: Nat Hum Behav. 2026 May 18;10(7):1327–39. doi: 10.1038/s41562-026-02472-x (PMC13388105; doi:10.1038/s41562-026-02472-x)
Supplement: Supplementary file 2 — Reporting summary [file 41562_2026_2472_MOESM2_ESM.pdf]

nature portfolio

Corresponding author(s): Deetje Iggena

Last updated by author(s): Mar 27, 2026

Reporting Summary

Nature Portfolio wishes to improve the reproducibility of the work that we publish. This form provides structure for consistency and transparency in reporting. For further information on Nature Portfolio policies, see our Editorial Policies and the Editorial Policy Checklist.

Statistics

For all statistical analyses, confirm that the following items are present in the figure legend, table legend, main text, or Methods section.

n/a	Confirmed	
☐	☒	The exact sample size (*n*) for each experimental group/condition, given as a discrete number and unit of measurement
☐	☒	A statement on whether measurements were taken from distinct samples or whether the same sample was measured repeatedly
☐	☒	The statistical test(s) used AND whether they are one- or two-sided<br>*Only common tests should be described solely by name; describe more complex techniques in the Methods section.*
☐	☒	A description of all covariates tested
☐	☒	A description of any assumptions or corrections, such as tests of normality and adjustment for multiple comparisons
☐	☒	A full description of the statistical parameters including central tendency (e.g. means) or other basic estimates (e.g. regression coefficient) AND variation (e.g. standard deviation) or associated estimates of uncertainty (e.g. confidence intervals)
☐	☒	For null hypothesis testing, the test statistic (e.g. *F*, *t*, *r*) with confidence intervals, effect sizes, degrees of freedom and *P* value noted<br>*Give P values as exact values whenever suitable.*
☒	☐	For Bayesian analysis, information on the choice of priors and Markov chain Monte Carlo settings
☒	☐	For hierarchical and complex designs, identification of the appropriate level for tests and full reporting of outcomes
☐	☒	Estimates of effect sizes (e.g. Cohen's *d*, Pearson's *r*), indicating how they were calculated

Our web collection on statistics for biologists contains articles on many of the points above.

Software and code

Policy information about availability of computer code

Data collection	For pointing tasks, Unity3D (version 2018.2.14f, Unity Technologies) and visual studio 2019 for C# code & for target placement task Psychtoolbox (Kleiner et al., 2007, software in MATLAB 9.5),in MATLAB (Mathworks, R2018b)
Data analysis	Statistical testing was conducted in R (v.4.4.1) and in MATLAB (Mathworks, R2018b).

For manuscripts utilizing custom algorithms or software that are central to the research but not yet described in published literature, software must be made available to editors and reviewers. We strongly encourage code deposition in a community repository (e.g. GitHub). See the Nature Portfolio guidelines for submitting code & software for further information.

Data

Policy information about availability of data

All manuscripts must include a data availability statement. This statement should provide the following information, where applicable:

- Accession codes, unique identifiers, or web links for publicly available datasets
- A description of any restrictions on data availability
- For clinical datasets or third party data, please ensure that the statement adheres to our policy

The data of this study is accessible at OSF: https://osf.io/sb65k/

Research involving human participants, their data, or biological material

Policy information about studies with human participants or human data. See also policy information about sex, gender (identity/presentation), and sexual orientation and race, ethnicity and racism.

Reporting on sex and gender	Data was collected on the self-reported sex. 102 female and 32 male. We included sex as a covariate in the generalized additive model but found no effect. Gender was not considered in this study.
Reporting on race, ethnicity, or other socially relevant groupings	We did not use race, ethnicity, or other socially relevant grouping.
Population characteristics	102 female and 32 male; Age range: 14 – 71; years of education range: 7 – 25.5
Recruitment	All participants were recruited via an online advertisement and on-site recruitment at Charité-Universitätsmedizin Berlin. Recruitment took place during the pandemic, so mainly Charité employees, their friends, acquiantances, and relatives were tested. As Charité is an organization with 20,000 employees, we had a wide range of social backgrounds in terms of occupation (pupil to senior consultant), education (years of education 7 – 25.5), and age (14-71). The first recruitment phase took place in August and September 2020 (58 participants) and the second recruitment phase from February 2022 to May 2023 (76 participants). In our study, women outnumbered men, which may have introduced bias and reduced the representativeness of the sample, thereby constraining the generalizability of the results.
Ethics oversight	All experimental procedures were conducted in accordance with the Declaration of Helsinki and were approved by the local ethics committee of Charité-Universitätsmedizin Berlin. All participants gave written informed consent.

Note that full information on the approval of the study protocol must also be provided in the manuscript.

Field-specific reporting

Please select the one below that is the best fit for your research. If you are not sure, read the appropriate sections before making your selection.

☐ Life sciences ☒ Behavioural & social sciences ☐ Ecological, evolutionary & environmental sciences

For a reference copy of the document with all sections, see nature.com/documents/nr-reporting-summary-flat.pdf

Behavioural & social sciences study design

All studies must disclose on these points even when the disclosure is negative.

Study description	Study on long-term spatial memory with a retrospective approach. Three different tasks were conducted to assess spatial memory across the ego- and allocentric spectrum. Quantitative measurements were used (deviation and distance).
Research sample	All participants were recruited via an online advertisement and on-site recruitment at Charité-Universitätsmedizin Berlin. Recruitment took place during the pandemic, so mainly Charité employees, their friends, acquiantances, and relatives were tested. As Charité is an organization with 20,000 employees, we had a wide range of social backgrounds in terms of occupation (pupil to senior consultant), education (years of education 7 – 25.5), and age (14-71). All participants had normal or corrected-to-normal vision, normal hearing, reported being in good health and denied neuropsychiatric disorders or substance abuse. But the sample is not representative of the general population as we did not recruit any children (< 14) and no seniors (> 71). Additionally, the sample was sex-imbalanced, with approximately three times as many female as male participants. Our primary goal was to recruit individuals with either prior exposure to the zoo or none. We did not select participants based on their demographic background.
Sampling strategy	As no comparable study on long-term memory has been conducted retrospectively so far, no sample size calculation was performed prior to the study. We considered a sample size of more than fifty to be sufficient to analyze the regression models and collected as many participants as possible during the recruitment periods to make the model as robust as possible. Our sample sizes are comparable to or larger than those reported in previous studies on long-term memory consolidation (Schmolck etal, 2000, Rubin et al. 1982).
Data collection	Unity3D (version 2018.2.14f, Unity Technologies) and Psychtoolbox (Kleiner et al., 2007, software in MATLAB 9.5) in MATLAB (Mathworks, R2018b) were used for data collection. The pointing tasks were performed with the immersive virtual reality headset HTC VIVE Pro Eye. The bird's eye view 2D map used for the tasks was programmed in MATLAB (Mathworks, R2018b) and visualized using Psychtoolbox (Kleiner et al., 2007). The task was presented on a Lenovo Thinkpad X1 Carbon laptop (14.0-inch screen). The experimenter was not blinded to the experimental conditions or hypotheses. However, this limitation is partially mitigated by the within-subject design, which reduces the risk of between-group expectancy effects. No one besides the study participant and the researcher were present.
Timing	The first recruitment phase took place in August and September 2020 (58 participants) and the second recruitment phase from February 2022 to May 2023 (76 participants). The prolonged break in data collection was due to the pandemic.
Data exclusions	Participants whose last visit to the zoo was more than 30 years ago were excluded, as only a few participants influenced the period

Data exclusions	after 30 years (n = 3). Individual measurements per participant were excluded if the respective destination was not yet open at the time of the last visit, e.g. the monkey house was only opened in 2000.
Non-participation	single measurement, no drop-out
Randomization	Allocation to conditions was not randomized, as group assignment was determined by participants' prior exposure to the zoo. We did not select participants based on their demographic background. To account for potential confounding, relevant demographic variables were used as covariates in statistical models.Zoo-visitor and zoo-naive controls conducted the same tasks but differed whether they had visited the zoo or not.

Reporting for specific materials, systems and methods

We require information from authors about some types of materials, experimental systems and methods used in many studies. Here, indicate whether each material, system or method listed is relevant to your study. If you are not sure if a list item applies to your research, read the appropriate section before selecting a response.

Materials & experimental systems

n/a	Involved in the study
☒	☐ Antibodies
☒	☐ Eukaryotic cell lines
☒	☐ Palaeontology and archaeology
☒	☐ Animals and other organisms
☒	☐ Clinical data
☒	☐ Dual use research of concern
☒	☐ Plants

Methods

n/a	Involved in the study
☒	☐ ChIP-seq
☒	☐ Flow cytometry
☒	☐ MRI-based neuroimaging

Plants

Seed stocks	*Report on the source of all seed stocks or other plant material used. If applicable, state the seed stock centre and catalogue number. If plant specimens were collected from the field, describe the collection location, date and sampling procedures.*
Novel plant genotypes	*Describe the methods by which all novel plant genotypes were produced. This includes those generated by transgenic approaches, gene editing, chemical/radiation-based mutagenesis and hybridization. For transgenic lines, describe the transformation method, the number of independent lines analyzed and the generation upon which experiments were performed. For gene-edited lines, describe the editor used, the endogenous sequence targeted for editing, the targeting guide RNA sequence (if applicable) and how the editor was applied.*
Authentication	*Describe any authentication procedures for each seed stock used or novel genotype generated. Describe any experiments used to assess the effect of a mutation and, where applicable, how potential secondary effects (e.g. second site T-DNA insertions, mosiacism, off-target gene editing) were examined.*
